# STAT3 expression correlates with prognosis of thymic epithelial tumors

**DOI:** 10.1186/1749-8090-8-92

**Published:** 2013-04-16

**Authors:** Chao Li, Zhou Wang, Yan Liu, Peng Wang, Runqi Zhang

**Affiliations:** 1 Department of Thoracic Surgery, Provincial Hospital affiliated to Shandong University, Jinan, P.R. China; 2 Department of Thoracic Surgery, Taian Central Hospital, Taian, P.R. China; 3 Department of Thoracic Surgery, Shandong Provincial Hospital, Shandong University, Jinan, 250012, P.R. China

**Keywords:** Thymic epithelial tumors, STAT3 protein, Immunohistochemistry, Prognosis

## Abstract

**Background:**

More and more evidences demonstrate the significance of Signal transducers and activators of transcription 3(STAT3) in oncogenesis and tumor development. However, little systematic researches have been reported on the correlation between STAT3 and thymic epithelial tumor (TET).

**Methods:**

Expression of STAT3 protein in 80 thymic epithelial tumors was detected by immunohistochemistry (IHC). The difference of STAT3 expression was compared by the *χ*^2^ test. Estimation of survival was calculated using the Kaplan-Meier method, and the statistical differences were analyzed using the Log-rank test.

**Results:**

Positive expression of STAT3 protein was significantly associated with Masaoka staging and WHO histological classification (P < 0.05), but not with age, gender, or tumor size. The rate of postoperative recurrence/metastasis was 33.33% in STAT3-positive tumors, compared with 4.55% in negative ones (P < 0.05). 5-year survival was significantly lower in STAT3-positive subjects (61.11%) than in negative ones (97.73%) (P < 0.01); In patients in Masaoka stage III or IV and WHO B3 or C, 5-year survival rate of subjects positive in STAT3 (35.00%, 35.00%) was statistically lower than that of the negative ones (92.31%, 91.67%). Cox regression analysis revealed that positive expression of STAT3 protein was an independent prognostic factor of thymic epithelial tumors (HR = 9.325, P = 0.044).

**Conclusion:**

Positive expression of STAT3 protein increases along with the rising malignant degree of thymic epithelial tumors. It may be considered as an independent prognostic parameter with good prognostic value to evaluate the possibility of recurrence/metastasis in patients with thymic epithelial tumor.

## Background

As frequently seen in the anterior mediastinum of adults, thymic epithelial tumor (TET) originates from thymic epithelial cells and comprises series of tumors different in pathology and biological behavior, including thymoma and thymic carcinoma. Now, the identification of benignancy and malignancy is still based on Masaoka staging and WHO histological classification [1], while the sensitivity of them is still unsatisfactory. The biological markers, therefore, are of great clinical importance to discriminate the benignant and malignant tumors.

In recent years, Janus protein tyrosine kinases/Signal transducers and activators of transcription (JAKs/STATs) has been paid more and more attention on its role in cellular signal transduction. Its downstream key molecules STAT3 is expressed abnormally and activated in multiple tumor tissues [2]. More and more evidences demonstrate the significance of STAT3 in oncogenesis and tumor development. However, little systematic researches have been reported on the correlation between TET and STAT3. In this study, we detected the expression of STAT3 in TETs through IHC, to explore its role in distinction between malignancy and benignancy and its correlation to prognosis.

## Methods

### General data

The present study was approved by the ethics committee of the University and was in compliance with the Helsinki Declaration. Additionally, the written informed consent was obtained from the family members of the patients.

Clinical data of patients with TET admitted to Provincial Hospital Affiliated to Shandong University from March 1999 to June 2009, who underwent surgical resection, were reviewed, and 80 patients with complete data were enrolled and evaluated in this retrospective study. Samples in this study were chosen from tumor tissues embedded in wax from Pathology Department, with another normal thymic tissue embedded in as the control. The study group contained 47 males and 33 females ranging in age from 19 to 70 years (mean = 46.5 y). No severe complications occurred during peri-operative period in any of the subjects. No patient underwent radiotherapy or chemotherapy prior to surgery. Complete resection was accomplished in 69 patients (86.3%) and palliative resection/biopsy was made in 11 (13.8%). The tumors were staged based on Masaoka’s clinical staging into stage I (n = 11), stage II (n = 14), stage III (n = 29) and stage IV (n = 4), and classified histologically according to WHO Histological Classification of Thymic Tumor 2004 into type A (n = 5), AB (n = 16), B1 (n = 10), B2 (n = 17), B3 (n = 10) and C (n = 22).

### Main reagents

Rabbit anti-human STAT3 polyclonal antibody and IHC kit were purchased from Santa Cruz, U.S.A; DAB color development kit was purchased from Dako; all other reagents were products above analytic pure, except where otherwise indicated.

### Immunohistochemistry

Slices were routinely dewaxed and hydrated through IHC SP. Antibodies were diluted 1:200 and underwent thermal repair. All other procedures were carried out strictly according to the instruction of IHC SP kit. The appearance of tan granules in plasma was considered as a positive staining of STAT protein, and the positive expression was scaled according to the ratio of positive cells. 10 high power fields (×400) were chosen randomly with 100 cancer cell in each to calculate the percentage of positive cells. A slice was determined to be negative if 0-10% of cells were tanned, or positive if tanned cells were more than 10%. All slices were determined by two pathologists with blindness, and a mutual agreement was arrived at through discussion made in case of any discrepancy.

### Follow up

The patients were followed up regularly, with records of sites and time point of tumor recurrence or metastasis. Patients who died from recurrence or metastasis were included into the survival analysis. The follow-up ended at the end of May 2010, with a median follow-up of 61.5 months (12-134 months). All patients were followed up, with a follow-up rate of 100%, through outpatient reexamination, telephone and letters.

### Statistical analysis

All statistic analyses were performed with SPSS 17.0 statistical software. The *χ*^2^ test was used to evaluate differences in STAT3 expression. Estimation of survival was calculated using the Kaplan-Meier method, and the statistical differences were analyzed using the Log-rank test. Cox regression multiple analysis was performed to determine the independent prognostic risk indicators. P < 0.05 was recognized as statistical significance.

## Results

### STAT3 expression in TET tissue and peritumoral normal thymic tissue

**Figure 1:**
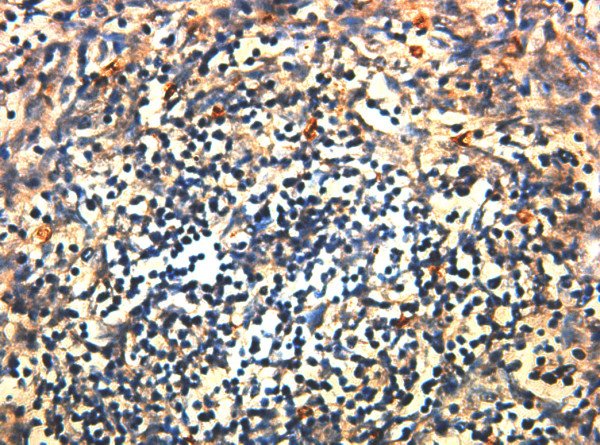
The expression of STAT3 in TET tissue (SP, ×400).

The positive expression of STAT3 in TET tissue appeared to be obvious tanned granules located in plasma (see Figure 1). 36 out of 80 patients (45%) were found positive in expression of STAT3 protein in TET tissue, while only 1 out of 20 (5%) peritumoral tissues was found positive. The difference was significant (p < 0.01) (see Table 1).

**Table 1:** The expression of STAT3 in TET tissue and peritumoral normal thymic tissue

	**STAT3protein(+)**	**STAT3protein(−)**	**Total**	**Positive rate**	***χ***^**2**^	***P *value**
TET tissues	36	44	80	45.00%		
Normal tissues	1	19	20	5.00%	10.982	P = 0.001
Total	38	62	100			

The difference of STAT3 expression in TET tissues and peritumoral normal thymic tissues is significant (P < 0 .01).

### STAT3 expression with clinicopathological characteristics of patients

Types and stages of STAT3 protein expression (percentage, ratio) were as follows: A (0/5, 0%); AB (4/16, 25%), B1(4/10, 40%), B2 (8/17, 44.44%), B3 (4/10, 40.00%), C (16/22, 72.73%), stage I (12/33, 36.36%), stage II (4/14, 25.57%), stage III (16/29, 55.17%), and stage IV (4/4, 100%). Statistics showed STAT3 correlated significantly to both WHO classification (*χ*^2^ = 13.742, P < 0.05) and Masaoka staging (*χ*^2^ = 8.623, P < 0.05), but not to age, gender and tumor mass (Table 2).

**Table 2:** Correlation of STAT3 protein expression to clinical pathology of patients

**Feature**	**N**	**STAT3 protein expression**		**Positive rate**	***χ*2**	**P value**
		**Positive**	**Negative**			
Age(year)					1.359	>0.05
<60	59	25	34	42.37%		
≥60	21	9	12	42.86%		
Gender					0.000	>0.05
Male	47	20	27	42.55%		
Female	33	14	19	42.42%		
WHO histologic subtype					13.742	<0.05
A	5	0	5	0		
AB	16	4	12	25.00%		
B1	10	4	6	40.00%		
B2	17	8	9	44.44%		
B3	10	4	6	40.00%		
C	22	16	6	72.73%		
Masaoka					8.623	<0.05
stage	33	12	21	36.36%		
Stage I	14	4	10	25.57%		
Stage II	29	16	13	55.17%		
Stage III	4	4	0	100%		
Stage IV						
Tumor size, d/cm					0.235	>0.05
≤5 cm	18	9	9	50.00%		
>5 cm	62	27	35	43.55%		

Statistics showed STAT3 correlated significantly to both WHO classification (*χ*2 = 13.742, P < 0.05) and Masaoka staging (*χ*2 = 8.623, P < 0.05), but not to age, gender and tumor mass.

### STAT3 expression with postoperative recurrence/metastasis

Recurrence/metastasis was found in 14 patients under follow-up, including 9 local recurrences and 5 remote metastases (including 2 patients with coincident local recurrence and remote metastasis), and the ratio of recurrence/metastasis was 17.50%. 12 recurrence/metastasis (33.33%) was noticed in patients positive in STAT3 protein expression, compared with 2 (4.55%) in those negative, with a significant difference (p < 0.05) (see Table 3).

**Table 3:** Correlation between STAT3 protein expression and post surgery recurrence/metastasis

	**Recurrence or metastasis**	**No recurrence or metastasis**	**Total**	***χ*2**	**P value**
STAT3 protein(+)	12	24	36		
STAT3 protein(−)	2	42	44		
Total	14	66	80	11.366	<0.01

The ratio of recurrence and metastasis between Stat3 protein positive and negative is significantly different (*χ*2 = 11.366, P < 0.01).

### STAT3 expression with prognosis of TET patients

**Figure 2:**
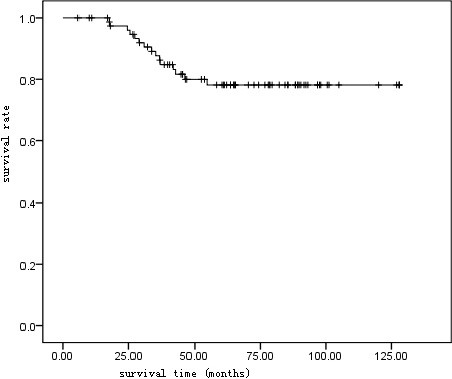
Kaplan-Meier overall survival curve of 80 TET patients.

**Figure 3:**
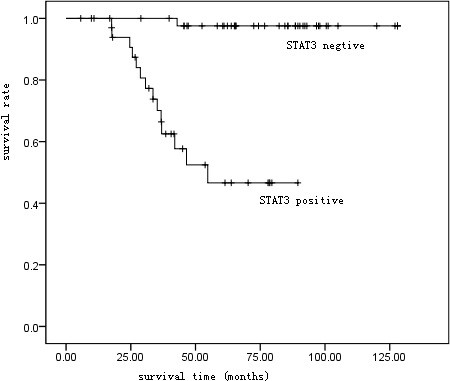
Kaplan-Meier survival curve of patients with STAT3 protein expression positive or negative.

Based on follow-up of 80 patients, the Kaplan-Meier survival curve suggested a 5-year survival rate of 81.25% (65/80) in all patients (Table 2 and Figure 2). The 5-year survival rate was 61.1% (22/36) in patients positive in STAT3 protein expression, and 97.73% (43/44) in those negative, with a statistical significance suggested by Log-rank test (p < 0.01) (Figure 3).

**Figure 4:**
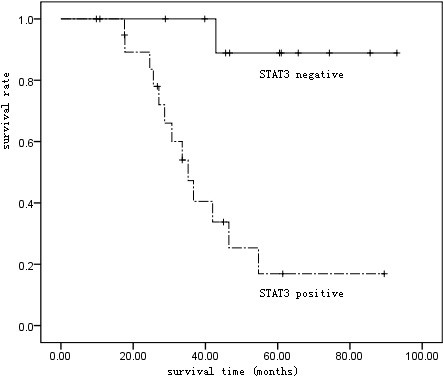
Kaplan-Meier survival curve of patients in WHO type B3 and C.

Our study involved 32 patients in type B3 or C, including 20 positive in STAT3 protein expression and 12 negative. The 5-year survival was 35.00% (7/20) in patients positive and 91.67% (11/12) in those negative, with significant difference proved by Log-rank test (p < 0.01) (Figure 4).

**Figure 5:**
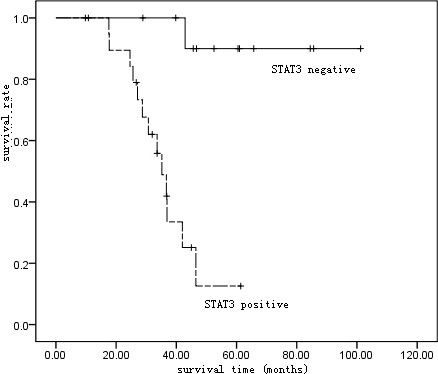
Kaplan-Meier survival curve of patients in Masaoka stage III and IV.

33 patients with thymic tumor in stage III or IV were included in our study, including 20 positive in STATS expression and 13 negative. 5-year survival was 35.00% (7/20) in patients positive and 92.31% (12/13) in those negative, with statistical difference demonstrated by Log-rank test (p < 0.01) (Figure 5).

### Multivariate analysis

Cox regression multiple analysis found the independent prognostic indicators including Masaoka staging (HR = 3.949, P = 0.016), complete excision (HR = 6.578, P = 0.015) and STAT3 protein expression (HR = 9.325, P = 0.044) for TET patients (Table 4).

**Table 4:** Results of Cox regression multiple analysis

	**B**	**SE**	**Wald**	**P**	**HR**	**95% CI for HR**
Age	−0.013	0.028	0.226	0.634	0.987	0.934~1.043
Gender	0.159	0.764	0.043	0.835	1.172	0.262~5.241
Mass size	0.014	0.281	0.003	0.960	1.014	0.585~1.760
STAT3 expression	2.233	1.110	4.043	0.044	9.325	1.058~82.207
Complete excision	1.884	0.773	5.936	0.015	6.578	1.445~29.939
WHO type	0.806	0.561	2.068	0.150	2.239	0.746~6.717
Masaoka stage	1.373	0.573	5.754	0.016	3.949	1.286~12.128

B: Regression coefficient; SE: Standard error; Wald: Wald value; P: P value;HR: Hazard ratio; CI: Confidence interval.

Cox regression multiple analysis found the independent prognostic indicators including Masaoka staging (HR = 3.949, P = 0.016), complete excision (HR = 6.578, P = 0.015) and STAT3 protein expression (HR = 9.325, P = 0.044) for TET patients.

## Discussion

Thymic epithelial tumor mainly includes thymoma and thymic carcinoma, and quite a few thymomas have certain degree of malignant features. It is a puzzle in clinical practice to evaluate precisely the malignancy degree and its prognosis.

Multiple oncogenes and tumor suppressor genes have been found correlated to oncogenesis, development and metastasis of thymic epithelial tumors (TET) [3]. STAT3, one of transcription factors under recent vigorous researches, is considered as an oncogene correlated closely to proliferation, differentiation, cellular apoptosis, immune escape, vascularization, invasion and metastasis in multiple malignancies [4]. Its abnormal expression can be found in many solid tumors and hematopoietic malignancies, such as leukemia, breast cancer, tumor in head and neck, skin cancer, brain malignant glioma, prostate cancer, lung cancer and liver cancer [5-11]. However, few researches have reported on relationship between STAT3 and thymic epithelial tumors.

We detected the positive expression of STAT3 protein in 45.00% of 80 TETs, compared with 5.00% in 20 para-tumorous normal tissues with significant significance (P < 0.01), implying the possible role of STAT3 protein in oncogenesis and development of TET and its potential value as a parameter in molecular biology in diagnosis of TET. Statistics showed expression of STAT3 protein correlated to both Masaoka staging (*χ*2 =13.742, P < 0.05) and WHO histological classification (*χ*2 = 8.623, P < 0.05). Our findings of the rising expression rate of STAT3 protein along with the increasing malignant degree of TET suggests that over expression of STAT3 may improve the proliferation and differentiation of thymic tumor cells and inhibit their apoptosis, and the excessively activated STAT3 may improve the oncogenesis and tumor development. No correlation was found by statistics between positive expression of STAT3 protein and gender, age, or tumor size of the patient. Overall 5-year survival in our 80 TET subjects was 81.25%, and patients’ positive in STAT3 protein had the significantly lower 5-year survival (61.11%) than the negative ones (97.73%). Follow up post surgery revealed a higher recurrence/metastasis rate (33.33%) in STAT3-positive patients than in the negative ones (4.55%), with statistical significance (P < 0.05).

Now, prognostic evaluation is mainly based on Masaoka staging and WHO classification in the clinical practice. Clinically, TET staged in III or IV belongs to the invasive TET, relatively poor in prognosis and low in probability of radical excision. However, thymic tumor in type A or AB generally have good prognosis even if they develop to the advanced stage, implying the discrepancy between prognosis and staging [12-14]. In our patients staged in III and IV, the 5-year survival were 35.00% and 92.31% respectively in STAT3-positive patients and the negative ones, with significant difference (P < 0.01). No death analysis was made for patients staged in I or II, for their relatively good prognosis and lower probability of death. Thymic tumor in type A based on 2004 WHO classification is considered a benignant tumor, but thymic cancers have been occasionally reported in type A [15]. B3 thymic tumor is a well differentiated thymic cancer, and type C is thymic cancer, both of which are highly malignant thymic epithelial tumor with poor prognosis [13,16]. In these two types of TETs in this study, the 5-year survival was 35.00% in STAT3-positive patients, in contrast to 91.67% in the negative ones, with significant difference (P < 0.01). All these findings revealed the significant difference in prognosis between patients differing in STAT3 expression level but having the same stage and same type based on Masaoka staging or WHO classification. Therefore, detection of STAT3 expression level may have vital importance to evaluate the prognosis of TET, especially precise for the highly malignant thymic epithelial tumor. Positive STAT3 means a bad prognosis, while the negative one means a good prognosis.

Despite the guiding significance of WHO classification and Masaoka staging in evaluation of the prognosis of thymic tumor, the definition of membrane infiltration has been made meaningless by the complete or partial absence of membrane in some of them. Moreover, no statistical difference in survival curve between Masaoka stage I and II, between WHO type A and B1, between B2 and B3, and between B3 and C [17-19] implies that it is still far from perfect of Masaoka staging and WHO classification to evaluate the prognosis. Our results from Cox multivariate regression supports STAT3 protein expression to be an independent prognostic factor for TET patients (HR = 9.325, P = 0.044), which helps establish a precise prognosis for patients with same staging and classification. Therefore STAT3 may be considered as a useful compensation for Masaoka staging and WHO classification.

We believe that it is necessary to carry out a further investigation on STAT3 gene, which may improve the precision of clinical diagnosis of thymic epithelial tumor, provide genetic diagnostic proofs, help the physicians make a precise forecast for their patients, provide new targets in genetically targeted therapy for thymic epithelial tumor patients, and have a great importance in genetic therapy for malignant thymic epithelial tumors not responsive to radiotherapy and/or chemotherapy.

## Conclusion

In summary, STAT3 may be used as an independent parameter to evaluate prognosis for patients with thymic epithelial tumor. As an indicator with good prognostic value in forecasting recurrence/metastasis, STAT3 may improve the diagnostic accuracy and provide better approaches in clinical practice for thymic epithelial tumor.

## Competing interests

The authors declare that they have no competing interests.

## Authors’ contributions

CL and WZ designed the study, collected the case information, drafted the manuscript, and performed statistical analysis. YL, PW and RZ collected the case information and performed statistical analysis. All authors read and approved the final manuscript.
